# Superiority of mucosal incision-assisted biopsy over ultrasound-guided fine needle aspiration biopsy in diagnosing small gastric subepithelial lesions: a propensity score matching analysis

**DOI:** 10.1186/s12876-020-1170-2

**Published:** 2020-01-21

**Authors:** Yosuke Minoda, Takatoshi Chinen, Takashi Osoegawa, Soichi Itaba, Kazuhiro Haraguchi, Hirotada Akiho, Akira Aso, Yorinobu Sumida, Keishi Komori, Haruei Ogino, Eikichi Ihara, Yoshihiro Ogawa

**Affiliations:** 1 Department of Medicine and Bioregulatory Science, Graduate School of Medical Sciences, Kyushu University, 3-1-1 Maidashi, Higashi-ku, Fukuoka, 812-8582 Japan; 2 Department of Gastroenterology, Clinical Research Institute, National Hospital Organization Kyushu Medical Center, Fukuoka, Japan; 3 Department of Gastroenterology, Kyushu Rosai Hospital, Fukuoka, Japan; 4 Department of Gastroenterology, Harasanshin Hospital, Fukuoka, Japan; 5 Department of Gastroenterology, Kitakyushu Municipal Medical Center, Fukuoka, Japan

**Keywords:** Subepithelial lesion, Mucosal incision-assisted biopsy, Ultrasound-guided fine needle aspiration biopsy

## Abstract

**Background:**

Gastric subepithelial lesions, including gastrointestinal stromal tumors, are often found during routine gastroscopy. While endoscopic ultrasound-guided fine-needle aspiration biopsy (EUS-FNAB) has been the gold standard for diagnosing gastric subepithelial lesions, alternative open biopsy procedures, such as mucosal incision-assisted biopsy (MIAB) has been reported useful. The aim of this study is to evaluate the efficacy of MIAB for the diagnosis of gastric SELs compared with EUS-FNAB.

**Methods:**

We retrospectively analyzed medical records of 177 consecutive patients with gastric SELs who underwent either MIAB or EUS-FNAB at five hospitals in Japan between January 2010 and January 2018. Diagnostic yield, procedural time, and adverse event rates for the two procedures were evaluated before and after propensity-score matching.

**Results:**

No major procedure-related adverse events were observed in either group. Both procedures yielded highly-accurate diagnoses once large enough samples were obtained; however, such successful sampling was more often accomplished by MIAB than by EUS-FNAB, especially for small SELs. As a result, MIAB provided better diagnostic yields for SELs smaller than 20-mm diameter. The diagnostic yields of both procedures were comparable for SELs larger than 20-mm diameter; however, MIAB required significantly longer procedural time (approximately 13 min) compared with EUS-FNAB.

**Conclusions:**

Although MIAB required longer procedural time, it outperformed EUS-FNAB when diagnosing gastric SELs smaller than 20-mm diameter.

## Background

Gastrointestinal stromal tumors (GISTs) and leiomyomas are the most common types of gastric subepithelial lesions (SELs) [1–3]. Many guidelines recommend histological evaluation of gastric SELs that have characteristics suggestive of GISTs [4–6]. Although endoscopic ultrasound-guided fine-needle aspiration biopsy (EUS-FNAB) has been the gold standard for such evaluation, obtaining large enough samples for histological analyses by using this technique is sometimes difficult, even with the recently-developed FNB needles and forward-viewing endoscopes.

Alternatively, “open biopsy” of SELs can be performed by partially removing the covering mucosa and exposing the lesion. Since the development of endoscopic submucosal dissection (ESD) for gastrointestinal neoplasms, ESD knives have increasingly been used to perform open biopsies of SELs [7–9]. We have named this biopsy technique, which was originally reported by Lee et al., [9] but which did not have a specific name, mucosal incision-assisted biopsy (MIAB) [10]. Our previous study suggested that MIAB is useful for SELs with intraluminal, but not extraluminal, growth patterns [11]. Our more recent study [11] suggested that MIAB and EUS-FNAB are of comparable usefulness for diagnosing SELs; however, the number of patients enrolled in that study (23 patients in MIAB group) was too small to enable evaluation of the efficacy of MIAB for SELs of differing sizes. Thus, in the current study, we collected medical records of a larger number of patients with SELs with intraluminal growth patterns and compared the usefulness, diagnostic yield, procedural time, and associated adverse events of MIAB and EUS-FNAB.

## Methods

### Patients

This was a retrospective comparative analysis of MIAB and EUS-FNAB using data for patients with gastric SELs with intraluminal growth patterns who underwent either MIAB or EUS-FNAB at five hospitals in Japan (Kyushu University Hospital, Kyushu Medical Center, Harasanshin Hospital, Kyushu Rosai Hospital, and Kitakyushu Municipal Medical Center) between January 2010 and January 2018. No patients who met the above criteria were excluded from the study. The decision to use MIAB or EUS-FNAB was left to the primary physician for each patient.

The intraluminal growth pattern of the SELs was confirmed by preoperative gastroscopy and EUS, and SEL size was measured on EUS images. All patients who underwent biopsy (either MIAB or EUS-FNAB) were suspected before biopsy of having tumors of mesenchymal origin, such as GISTs, leiomyomas, schwannomas, and glomus tumor, on the basis of EUS findings.

### MIAB and EUS-FNAB procedures

For MIAB, to lift the mucosa covering the SEL and to create a safer incision, normal saline or glycerol supplemented with diluted epinephrine was injected into the submucosal layer above the lesion. The target mucosal and submucosal tissues were incised with an endoscopic submucosal dissection knife (Needle knife, Olympus, Tokyo, Japan; Flush knife, Fuji Film, Tokyo, Japan) using electrosurgical current generated by a high-frequency power supply (ICC or VIO300D; ERBE, Tubingen, Germany). After exposing the lesion, tissue samples were obtained by biopsy forceps (Radial jaw, Boston Scientific, Natick, MA, USA). Approximately 3–7 biopsy samples were obtained from each lesion.

For EUS-FNAB, 19- to 25-gauge FNAB needles (described later) were used to obtain SEL samples through the mucosa under the guidance of oblique-view EUS imaging (GF-UCT 260; Olympus). Rapid on-site evaluation (ROSE) by cytologists or pathologists was performed for all EUS-FNAB procedures. Half of each FNAB sample was used for ROSE and the other half reserved for later histological examination. The procedures were repeated a maximum of six times until either the biopsy team considered they had obtained enough samples for histology, for example, because the cytologists/pathologists had seen numerous spindle cells, indicating that the needle had reached a GIST, or the team decided to end the procedure due to difficulties in sample collection.

All samples in both groups were later evaluated histologically by pathologists. All patients were monitored daily for symptoms and signs of hematomesis and hematochezia.

### Comparison between MIAB and EUS-FNAB

Three aspects of MIAB and EUS-FNAB were compared: diagnostic yield, procedural time, and adverse event rate. The procedural time was defined as the time from start to finish of the biopsy procedures. The data were collected from the operational records of patients. Major bleeding was defined as a ≥ 2 g/dL drop in blood hemoglobin.

### Comparison between EUS-FNA and EUS-FNB

The following needles were categorized as FNA and FNB needles: FNA; SonoTip, Mediglobe GmbH, Rosenheim, Germany; Expect, Boston Scientific; Ez-shot, Olympus, Tokyo, Japan; and FNB: Echo-Tip Procore, Cook Medical, Bloomington, USA; Acquire, Boston Scientific. The diagnostic yields achieved by FNA and FNB needles were compared.

### Statistical analysis

**Fig. 1:**
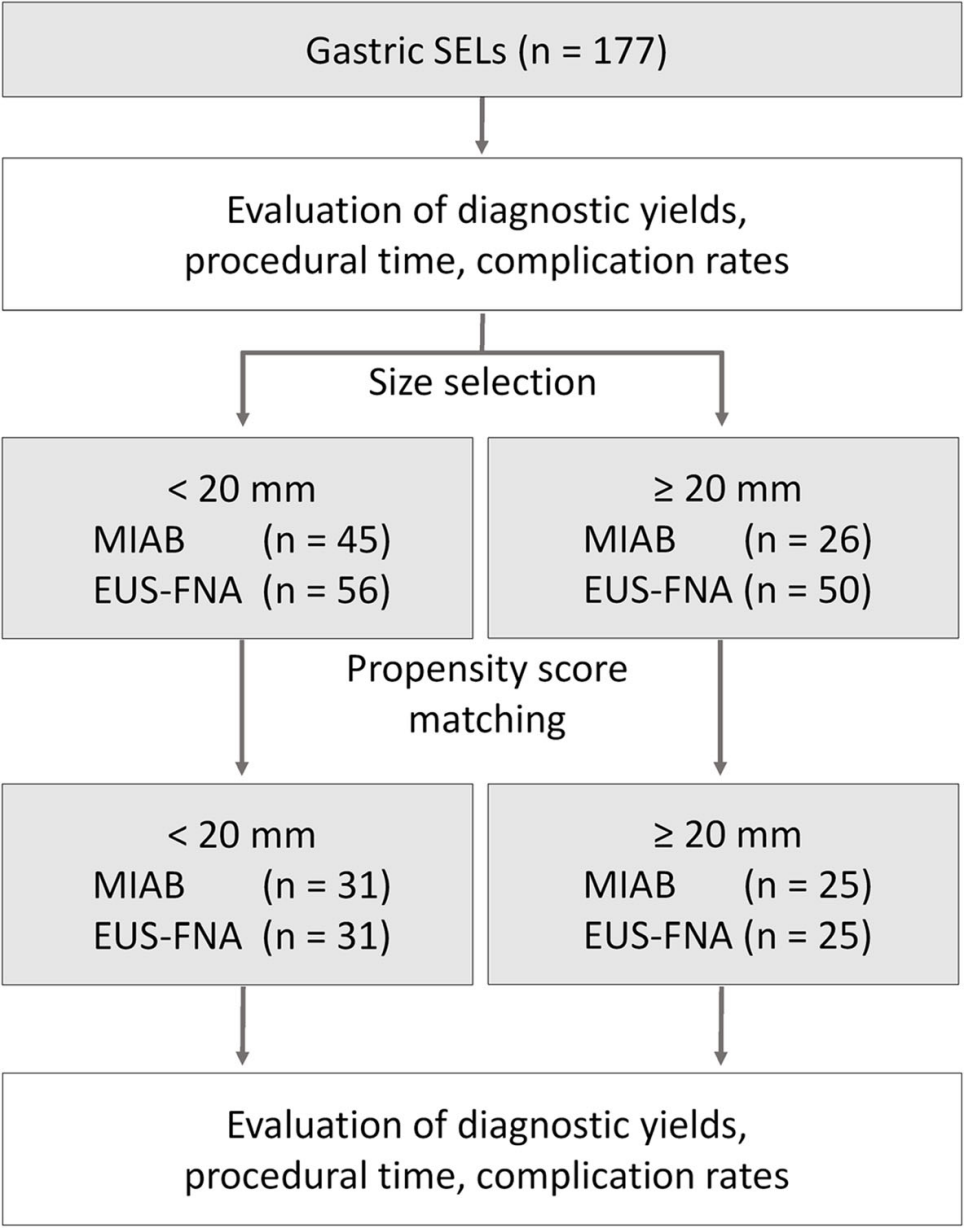
Summary of the study protocol

All statistical analyses were performed using the JMP software program version 13.0 (SAS Institute, Cary, NC, USA). Comparisons between MIAB and EUS-FNAB were performed before and after propensity-score matching of the lesion sizes in the two groups (Fig. 1). The chi-squared test or Fisher’s exact test were used to compare the categorical data (patients’ characteristics, lesion locations, and histology types). Student’s *t*-test was used to compare continuous data (age, lesion size, and procedural time) before propensity-score matching. We used a multivariate logistic regression analysis to evaluate the relationship between the diagnostic yield and lesion size. After propensity-score matching, Student’s paired *t*-test was used to compare continuous data in the two groups. *P* < 0.05 indicated statistical significance, for all tests.

To estimate propensity scores, lesion sizes (mm) were entered as independent variables into a multivariate logistic regression model. This model yielded an area under the receiver operating characteristic curve score of 0.67. Once the propensity scores were estimated, we matched patients in the two groups by setting calipers, using the stringency scores in the JMP software program, at a width equal to a distance of 0.2 from the standard deviation of the logit of the propensity score, without replacement. The effect of matching was evaluated in terms of the absolute standardized difference.

## Results

### Comparisons between MIAB and EUS-FNAB

A total of 177 SELs from 177 consecutive patients (male, *n* = 87; female, *n* = 90) were included in this study. The characteristics of the patients and lesions in both groups are summarized in Table 1. Seventy-one patients underwent MIAB, and 106 underwent EUS-FNAB. There were no significant differences between the groups in sex, age, lesion size, lesion location, or histological type. No procedure-related adverse events, including late-onset bleeding after discharge from hospital, major bleeding, or gastric perforation, occurred in either group. The success rate of tissue sampling was higher with MIAB than with EUS-FNAB (95.6% vs. 86.8%, respectively; *P* = 0.047). Accordingly, the diagnostic yield of MIAB was significantly higher than that of EUS-FNAB (94.3% vs. 79.2%, respectively; *P* = 0.013). However, MIAB took significantly longer to perform than EUS-FNAB (31.5 min vs. 21 min, respectively; *P* < 0.0001). Among the SELs successfully diagnosed by either MIAB or EUS-FNAB, 102 were diagnosed as GISTs, 69 of which were surgically resected in one of the five participating hospitals; all were confirmed to be GISTs.

**Table 1:** Patient and lesion characteristics

	MIAB group	EUS-FNAB group	*P* value
Number of patients	71	106	
Gender; male/female	31/40	56/50	n.s. (*P* = 0.23)
Age; median & range	62 (27–84)	63 (27–87)	n.s. (*P* = 0.95)
Lesion size (mm); median & range	19.6 (8.8–48)	20.0 (9–63)	n.s. (*P*= 0.096)
Number of lesions in each gastric location			n.s. (*P*= 0.61)
Upper stomach	40	66	
Middle stomach	18	26	
Lower stomach	13	14	
Procedural time (min); median & range	31.5 (9–160)	21.0 (8–55)	*P* < 0.0001
Success rate of tissue sampling	95.6% (68/71)	86.8% (92/106)	*P* = 0.047
Diagnostic yield	94.3% (67/71)	79.2% (84/106)	*P* = 0.013
Complication rate	0% (0/71)	0% (0/106)	n.s (*P* = 1.0)
Number of lesions of each histology type			n.s. (*P* = 0.053)
GIST	53.5% (38/71)	60.4% (64/106)	
Leiomyoma	25.3% (18/71)	11.3% (12/106)	
Schwannoma	2.8% (2/71)	3.8% (4/106)	
Aberrant pancreas	8.5% (6/71)	2.8% (3/106)	
Glomus tumor	1.4% (1/71)	–	
Lipoma	1.4% (1/71)	–	
Inflammatory change	1.4% (1/71)	–	
Renal cell carcinoma	–	0.9% (1/106)	
Matching rate of pre- and post-operative diagnoses	100% (35/35)	100% (34/34)	n.s. (*P* = 1.0)

### The effects of SEL size and location on diagnostic yields of EUS-FNAB and MIAB

As shown in Table 2, the diagnostic yield with EUS-FNAB for SELs < 20-mm diameter was significantly lower than with SELs ≥20-mm diameter (88.0% vs 71.4%, respectively; *P* = 0.048). In contrast, the diagnostic yield with MIAB was not affected by lesion size (92.3% vs 93.3%, < 20-mm vs ≥ 20-mm diameter, respectively; *P* = 0.51). The diagnostic yields for samples from the upper/middle/lower parts of the stomach were 92.5%/88.9%/100% with MIAB and 84.5%/76.9%/57.1% with EUS-FNAB. SEL location did not affect the diagnostic yield with either procedure (*P =* 0.48 with MIAB and *P =* 0.067 with EUS-FNAB).

**Table 2:** Relationships between lesion size and location, and diagnostic yields with MIAB and EUS-FNAB

	MIAB group	*P* value	EUS-FNAB group	*P* value
Lesion sizes and diagnostic yield	≥ 20 mm: 92.3%	n.s. (*P* = 0.51)	≥ 20 mm: 88.0%	*P* = 0.048
	< 20 mm: 93.3%		< 20 mm: 71.4%	
Lesion locations and diagnostic yield	Upper: 92.5%	n.s. (*P* = 0.48)	Upper: 84.5%	n.s. (*P* = 0.067)
	Middle: 88.9%		Middle: 76.9%	
	Lower: 100%		Lower: 57.1%	

### Comparison of MIAB and EUS-FNAB before and after propensity-score matching

Because the initial analyses suggested the superiority of MIAB over EUS-FNAB, especially for diagnosing SELs < 20-mm diameter, the SELs were divided into two groups (≥ 20-mm and <  20-mm diameter) and MIAB and EUS-FNAB compared (Fig. 1, Tables 3 and 4). For this comparison, lesion sizes were matched between the MIAB and EUS-FNA groups using propensity-score matching analysis. As shown in Tables 3 and 4, after matching propensity scores on the basis of lesion size, there was no difference between the two groups in patient characteristics or sizes and locations of lesions. The procedural times for MIAB and EUS-FNAB for SELs ≥20-mm diameter were 32 min and 20.5 min, respectively, whereas procedural times for MIAB and EUS-FNAB for SELs < 20-mm diameter were 31 min and 20 min, respectively. MIAB took significantly longer to perform (on average, 12–14 min longer) than EUS-FNAB, regardless of lesion size. For SELs ≥20-mm diameter, the success rate of tissue sampling and diagnostic yields did not differ significantly between the two procedures (*P* = 0.55) (Table 3). However, for SELs < 20-mm diameter, MIAB yielded a significantly higher successful diagnosis rate than did EUS-FNAB (93.5% vs. 61.2%, respectively; *P* = 0.011) (Table 4). The results of the analysis after matching the propensity scores based on lesion sizes and locations are shown in Additional file 1: Table S1. Again, the procedural time was significantly longer for MIAB than EUS-FNAB for all sizes of SELs. Both the success rate of tissue sampling and the diagnostic yield were higher with MIAB than with EUS-FNAB for SELs < 20 mm.

**Table 3:** Comparison of MIAB and EUS-FNAB in diagnosing SELs ≥20-mm diameter (using the matching factor of lesion size)

	Before matching			After matching		
	MIAB group	EUS-FNAB group	*P* value	MIAB group	EUS-FNAB group	*P* value
Number of patients	26	50	n.s. (*P* = 0.051)	25	25	n.s. (*P* = 1.0)
Gender; male/female	10/16	19/31	n.s. (*P* = 0.051)	10/15	11/14	n.s. (*P* = 0.77)
Age; median & range	62.5 (24–79)	63.5 (28–78)	n.s. (*P* = 0.88)	62 (24–79)	68 (36–77)	n.s. (*P* = 0.27)
Lesion size (mm); median & range	26.2 (20–48)	28 (20–63)	*P* = 0.040	25 (20–36)	24 (20–36)	n.s. (*P* = 0.95)
Number of lesions in each gastric location			n.s. (*P* = 0.98)			n.s. (*P* = 0.93)
Upper stomach	16	32		15	16	
Middle stomach	5	9		5	5	
Lower stomach	5	9		5	4	
Procedural time (min); median & range	32 (9–70)	22.5 (8–55)	*P* = 0.043	32 (9–70)	20.5 (8–41)	*P* = 0.018
Success rate of tissue sampling	96.1% (25/26)	90.0% (45/50)	*P* = 0.062	96.0% (24/25)	96.0% (24/25)	n.s. (*P* = 1.0)
Diagnostic yield	92.3% (24/26)	88.0% (44/50)	n.s. (*P* = 0.56)	96.0% (24/25)	96.0% (24/25)	n.s. (*P* = 1.0)
Complication rate	0% (0/26)	0% (0/50)	n.s. (*P* = 1.0)	0% (0/25)	0% (0/25)	n.s. (*P* = 1.0)
Number and frequency of lesions of each histology type			n.s. (*P* = 0.12)			n.s. (*P* = 0.091)
GIST	65.3% (17/26)	64.0% (32/50)		64% (16/25)	88% (22/25)	
Leiomyoma	23.1% (6/26)	16.0% (8/50)		24% (6/25)	4% (1/25)	
Schwannoma	–	6.0% (3/50)		–	4% (1/25)	
Aberrant pancreas	8.0% (2/26)	–		8% (2/25)	–	
Renal cell carcinoma		2.0% (1/50)		–	–

**Table 4:** Comparison of MIAB and EUS-FNAB in diagnosing SELs < 20-mm diameter (using the matching factor of lesion size)

	Before matching			After matching		
	MIAB group	EUS-FNAB group	*P* value	MIAB group	EUS-FNAB group	*P* value
Number of patients	45	56	n.s. (*P* = 0.84)	31	31	n.s. (*P* = 1.0)
Gender; male/female	21/24	25/31	n.s. (*P* = 0.84)	14/17	13/18	n.s. (*P* = 0.80)
Age; median & range	62.0 (27–84)	62.0 (27–87)	n.s. (*P* = 0.98)>	62.0 (27–82)	64.0 (27–83)	n.s. (*P* = 0.77)
Lesion size (mm); median & range	15.0 (8.8–19.8)	16.0 (9.0–19.8)	n.s. (*P* = 0.84)	17 (8.8–19.8)	15 (9–19.8)	n.s. (*P* = 0.99)
Number of lesions in each gastric location			n.s. (*P* = 0.41)			n.s. (*P* = 0.92)
Upper stomach	24	34		18	18	
Middle stomach	13	17		8	9	
Lower stomach	8	5		5	4	
Procedural time (min); median & range	31 (10–160)	20 (9–49)	*P* < 0.001	31 (10–160)	20 (10–49)	*P* = 0.0093
Success rate of tissue sampling	97.8% (44/45)	85.7% (48/56)	*P* = 0.34	93.5% (29/31)	61.3% (19/31)	*P* = 0.011
Diagnostic yield	93.3% (42/45)	71.4% (40/56)>	*P* = 0.005	93.5% (29/31)	61.3% (19/31)	*P* = 0.011
Complication rate	0% (0/45)	0% (0/56)	n.s. (*P* = 1.0)	0% (0/31)	0% (0/31)	n.s. (*P* = 1.0)
Number and frequency of lesions of each histology type			n.s. (*P* = 0.066)			n.s. (*P* = 0.14)
GIST	46.7% (21/45)	57.1% (32/56)		48.4% (15/31)	42.0% (13/31)	
Leiomyoma	26.7% (12/45)	8.9% (5/56)		25.8% (8/31)	12.9% (4/31)	
Schwannoma	4.4% (2/45)	1.7% (1/56)		6.5% (2/31)	–	
Aberrant pancreas	8.9% (4/45)	3.4% (2/56)		6.5% (2/31)	6.5% (2/31)	
Glomus tumor	2.2% (1/45)	–		–	–	
Lipoma	2.2% (1/45)	–		3.2% (1/31)	–	
Inflammatory change	2.2% (1/45)	–		3.2% (1/31)	–	

### Comparison between EUS-FNA, EUS-FNB, and MIAB

Because previous reports have suggested that the more recently developed FNB needles are superior to FNA needles for collecting samples, especially for pancreatic lesions [12–14], the diagnostic yields of the two types of needles were compared with yields for MIAB. FNA needles were used for 69 and FNB needles for 37 of the 106 patients who underwent EUS-FNAB. For SELs ≥20-mm diameter, EUS-FNA and EUS-FNB were performed in 33 and 17 patients, respectively, whereas for SELs < 20-mm diameter, EUS-FNA and EUS-FNB were performed in 36 and 20 patients, respectively.

**Fig. 2:**
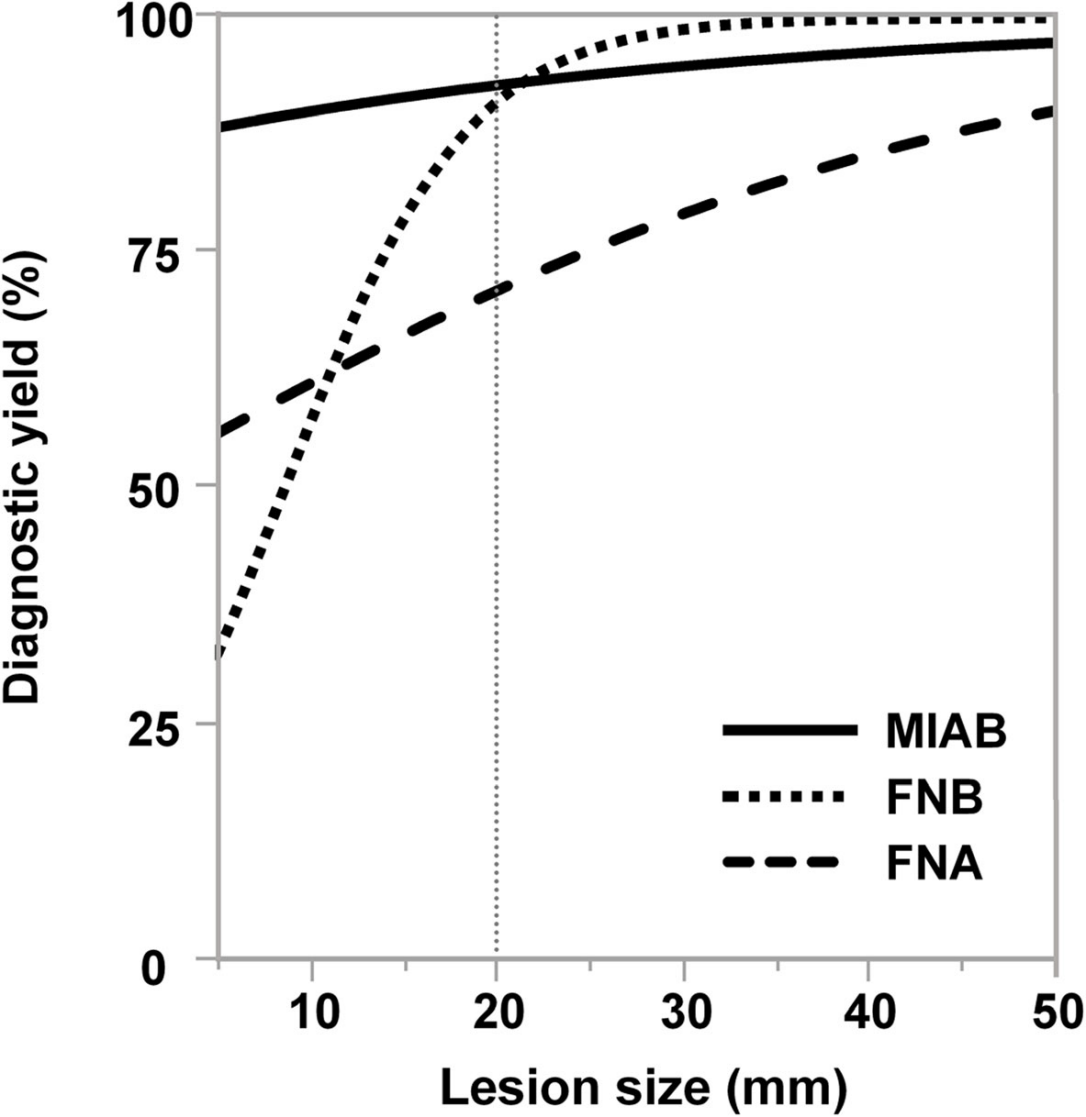
Relationships between the lesion sizes and diagnostic yields. The regression curves for MIAB, EUS-FNA, EUS-FNB were generated from the data shown in Tables 1, 2, 3, 4, 5 and 6

For SELs ≥20-mm diameter, the diagnostic yield of EUS-FNB (100%) was significantly higher than that of EUS-FNA (80.7%) (*P* = 0.041), but comparable to that of MIAB (96%) (*P* = 0.38). However, for SELs < 20-mm diameter, the diagnostic yields of EUS-FNA and EUS-FNB did not differ significantly (68.7 and 77.8%, respectively) (*P* = 0.065). For this size of SELs, MIAB outperformed both EUS-FNA and EUS-FNB (Tables 5, 6, Fig. 2).

**Table 5:** Patient and lesion characteristics, who underwent EUS-FNA and EUS-FNB

	EUS-FNA group	EUS-FNB group	*P* value
Number of patients	69	37	
Gender; male/female	33/36	23/14	n.s. (P = 0.16)
Age; median & range	62 (27–87)	65 (36–78)	n.s. (*P* = 0.42)
Lesion size (mm); median & range	20.0 (9–58)	21.0 (10–63)	n.s. (*P* = 0.94)
Number of lesions in each gastric location			n.s. (*P* = 0.22)
Upper stomach	41	25	
Middle stomach	16	10	
Lower stomach	12	2	
Procedural time (min); median & range	22 (9–55)	20 (8–49)	n.s. (*P* = 0.76)
Success rate of tissue sampling	84.1% (58/69)	91.9% (34/37)	n.s. (*P* = 0.26)
Diagnostic yield	73.9% (51/69)	89.2% (33/37)	n.s. (*P* = 0.065)
Complication rate	0% (0/69)	0% (0/37)	n.s. (*P* = 1.0)

**Table 6:** Comparison of EUS-FNA and EUS-FNB in diagnosing SELs

	SELs < 20 mm			SELs ≥ 20 mm		
	EUS-FNA group	EUS-FNB group	*P* value	EUS-FNA group	EUS-FNB group	*P* value
Number of patients	38	18		31	19	
Gender; male/female	16/22	9/9	n.s. (*P* = 0.58)	17/14	14/5	n.s. (*P* = 0.18)
Age; median & range	62.5 (27–87)	60.5 (38–77)	n.s. (*P* = 0.95)	61 (28–77)	66 (36–78)	n.s. (*P* = 0.20)
Lesion size (mm); median & range	15 (9–19.8)	16 (10–19.8)	n.s. (*P* = 0.35)	30 (20–58)	26 (20–63)	n.s. (*P* = 0.86)
Number of lesions in each gastric location			n.s. (*P* = 0.27)			n.s. (*P* = 0.94)
Upper stomach	22	12		19	13	
Middle stomach	11	6		5	4	
Lower stomach	5	0		7	2	
Procedural time (min); median & range	20 (9–37)	23 (11–49)	n.s. (*P* = 0.18)	25 (9–55)	19 (8–41)	n.s. (*P* = 0.41)
Success rate oftissue sampling	79.0% (30/38)	83.3% (15/18)	n.s. (*P* = 0.70)	90.3% (28/31)	100% (19/19)	n.s. (*P* = 0.16)
Diagnostic yield	68.4% (26/38)	77.8% (14/18)	n.s. (*P* = 0.47)	80.1% (25/31)	100% (19/19)	*P* = 0.041
Complication rate	0% (0/38)	0% (0/18)	n.s. (*P* = 1.0)	0% (0/31)	0% (0/19)	n.s. (*P* = 1.0)

## Discussion

To diagnose GISTs, immunohistochemical staining for several antigens, such as c-Kit, DOG1, and S-100, is necessary [4, 15–18]. Obtaining samples large enough to perform several immunohistochemical evaluations is sometimes very difficult when performing EUS-FNAB, especially when the lesion is small [19]. This leads to failure in making a diagnosis despite time-consuming procedures and on-site evaluations by pathologists. The reported diagnostic yield of EUS-FNAB for small gastric SELs is 62–82% [19, 20].

In the current study, we showed the superiority of MIAB over EUS-FNAB for diagnosing gastric SELs with intraluminal growth < 20-mm diameter. Our findings are partially consistent with a previous study that reported comparable diagnostic yields with MIAB and EUS-FNAB for gastric SELs [7]. However, in that study the lesions were not classified into small and large groups; MIAB is especially useful for obtaining samples from small SELs. Although metastasis or invasion of GISTs < 20 mm diameter is considered very rare [21, 22], many guidelines recommend surgical resection of GISTs, regardless of the lesion size. We have encountered a patient with metastasis from a GIST of approximately 15 mm diameter [23]. Improving biopsy skills for such small SELs is necessary. MIAB does not require EUS during biopsy, nor does it require on-site evaluation by cytologists or pathologists. With MIAB, it is immediately evident whether samples sufficient for histological evaluation have been obtained. Therefore, MIAB could be preferable considering the possibility of diagnostic failure following FNAB and situations where EUS systems are unavailable. Very similar open biopsy techniques, such as single-incision needle-knife biopsy (SINK) and unroofing biopsy have also been reported [24–26]. These procedures may have advantages similar to those of MIAB.

The designs of aspiration needles have been modified to enable collection of larger biopsy samples, including development of the so-called fine needle biopsy (FNB) needles. Although FNB needles are reportedly superior to conventional FNA needles for the diagnosis of pancreatic lesions, their usefulness for diagnosis of gastric SELs is controversial [27–29]. Our findings suggest that the diagnostic yields with EUS-FNA, EUS-FNB, and MIAB are comparable for SELs ≥20-mm diameter. For SELs < 20-mm diameter, our results are consistent with those reported for pancreatic lesions, where FNB needles outperformed FNA needles. However, MIAB outperformed both FNA and FNB needles in terms of diagnostic yield.

The strategies for the treatment of SELs with diameters within the range of 20–50-mm slightly differ among guidelines. The Japanese guidelines recommend biopsy for such SELs, whereas the European and American guideline recommends either performing biopsy or directly resecting the lesion [4–6]. In our study, despite the fact that all patients who underwent biopsy were suspected of having tumorous lesions on the basis of EUS findings, a few lesions turned out to be non-tumorous, such as the aberrant pancreas and inflammatory reactions. Because a few SELs show atypical EUS findings, we think biopsy is the preferable approach than direct surgery for SELs within 20–50-mm diameter. The diagnostic yield with MIAB for SELs within this range were comparable to that with EUS-FNAB. However, considering the fact that MIAB takes longer to perform and is only effective for SELs with intraluminal growth, EUS-FNAB would remain the standard biopsy procedure for SELs with diameters within this range.

Our study also revealed that MIAB and EUS-FNAB are very safe techniques. No major or minor adverse events were reported in our hospitals. Although MIAB uses skills and devices developed for endoscopic submucosal dissection, the adverse event rate following MIAB was much lower than that reported for endoscopic submucosal dissection. The incidence of bleeding in patients undergoing endoscopic submucosal dissection for gastric mucosal tumors is reportedly 2–15% [30–35] . The lower rate with MIAB is likely because this procedure requires only a partial incision into the normal mucosa covering the SEL, whereas with endoscopic submucosal dissection an entire tumor of epithelial origin (i.e., adenoma or adenocarcinoma) is resected. Such epithelial tumors are usually fed by thick vessels from the submucosal layer; thus, endoscopic submucosal dissection requires transecting those vessels, which could lead to major or late-onset bleeding. Our data suggest that MIAB is a safe and reliable approach; however, one concern about this procedure is that it exposes tumor cells of GISTs by disrupting the fibrous pseudocapsule within which those tumor cells are usually encapsulated. EUS-FNAB also requires piercing of the pseudocapsule; however, the area that is disrupted (a pinhole) is much smaller than that with MIAB. Thus, in MIAB, more cells could be released into the gastric lumen from the biopsied area. It is unlikely that such freed tumor cells would attach to and grow in other parts of the gastrointestinal tract; however, when the stomach is perforated during an unsatisfactory procedure and gastric contents are released into the abdominal cavity, the chances of tumor seeding may be increased. Although we have not encountered MIAB-associated perforation or tumor seeding, great care should be taken to avoid this. In particular, MIAB should be performed only on lesions that are visible by conventional endoscopy.

As to limitations, this study had a retrospective design. Thus, our findings require confirmation by a prospective randomized trial; however, our data provide reasonable support for open biopsy as an option for obtaining samples from gastric SELs.

## Conclusions

In conclusion, although MIAB takes approximately 13 min longer to perform than EUS-FNAB, MIAB provides significantly better diagnostic yields for gastric SELs smaller than 20-mm diameter with intraluminal growth, regardless of their location. MIAB may be a good option for diagnosing small gastric SELs. In our study, the diagnostic yields with MIAB and EUS-FNAB were comparable for gastric SELs ≥20-mm diameter. Considering the shorter procedural time required, EUS-FNAB should remain the standard for diagnosing larger lesions.

## Supplementary information

**Additional file 1: Table S1.** Comparison of MIAB and EUS-FNAB in diagnosing SELs < 20-mm diameter (using the matching factors of lesion size and location).

## Data Availability

The datasets used and analyzed during the current study are available from the corresponding author upon reasonable request.

## Abbreviations

EGD: Esophagogastroduodenoscopy
EMR: Endoscopic mucosal resection
ESD: Endoscopic submucosal dissection
EUS-FNAB: Endoscopic ultrasound-guided fine needle aspiration/ biopsy
FNB: Fine needle biopsy
GIST: Gastrointestinal stromal tumor
MIAB: Mucosal incision-assisted biopsy
PPI: Proton pump inhibitor
ROSE: Rapid on-site evaluation
SEL: Subepithelial lesion
SINK: Single-incision needle-knife biopsy
