# Supplementary material for: Superiority of mucosal incision-assisted biopsy over ultrasound-guided fine needle aspiration biopsy in diagnosing small gastric subepithelial lesions: a propensity score matching analysis
Source: BMC Gastroenterol. 2020 Jan 21;20:19. doi: 10.1186/s12876-020-1170-2 (PMC6975081; doi:10.1186/s12876-020-1170-2)
Supplement: Supplementary file 1 — Additional file 1: Table S1. Comparison of MIAB and EUS-FNAB in diagnosing SELs < 20-mm diameter (using the matching factors of lesion size and location). [file 12876_2020_1170_MOESM1_ESM.docx]

Additional file 1: Table S1.

|  | SELs < 20mm (after matching) | | | SELs ≥ 20mm (after matching) | | |
| --- | --- | --- | --- | --- | --- | --- |
|  | MIAB  group | EUS-FNAB  group | *P* value | MIAB  group | EUS-FNAB group | *P* value |
| Number of patients | 42 | 42 | n.s. (*P* = 1.0) | 25 | 25 | n.s. (*P* = 1.0) |
| Gender; male/female | 19/23 | 15/27 | n.s.  (*P* = 0.37) | 10/15 | 12/13 | n.s.  (*P* = 0.57) |
| Age; median & range | 62.0  (27–84) | 64.5  (27–87) | n.s.  (*P* = 0.40) | 62  (24–79) | 66  (28–77) | n.s.  (*P* = 0.77) |
| Lesion size (mm);  median & range | 15  (8.8–19.8) | 15  (9–19.8) | n.s.  (*P* = 1.0) | 25  (20–36) | 24  (20–36) | n.s.  (*P* = 0.86) |
| Number of lesions  in each gastric location |  |  | n.s.  (*P* = 1.0) |  |  | n.s.  (*P* = 0.94) |
| Upper stomach | 24 | 24 |  | 15 | 14 |  |
| Middle stomach | 13 | 13 |  | 5 | 6 |  |
| Lower stomach | 5 | 5 |  | 5 | 5 |  |
| Procedural time (min);  median & range | 34  (10–160) | 25  (9–35) | ***P* = 0.0037** | 32  (8–70) | 22.5  (8–50) | ***P* = 0.018** |
| Success rate of  tissue sampling | 100%  (42/42) | 85.7%  (36/42) | ***P* = 0.011** | 88.0%  (22/25) | 92.0%  (23/25) | n.s.  (*P* = 0.14) |
| Diagnostic yield | 92.7%  (39/42) | 66.7%  (28/42) | ***P* = 0.0028** | 96.0%  (24/25) | 92.0%  (23/25) | n.s.  (*P* = 0.63) |
| Complication rate | 0% (0/42) | 0% (0/42) | n.s. (*P* = 1.0) | 0% (0/25) | 0% (0/25) | n.s. (*P* = 1.0) |
